# Comparison of Clinical Effectiveness of Drug-Coated Balloons versus Percutaneous Transluminal Angioplasty in Arteriovenous Fistulae Maturation Failure: A Multicenter Prospective Study

**DOI:** 10.1007/s00270-025-04336-9

**Published:** 2026-01-30

**Authors:** Leonardo Harduin, Leonardo Cortizo, Márcio Gomes Filippo, Thiago Almeida Barroso, Julia Bandeira Guerra, Renata Silveira Mello, Carlos Alexandre Rosa Gama, Brenda Kaori Ishiy Ozima, Brunno Ribeiro Vieira, Paulo Eduardo Ocke Reis, Jorge Paulo Strogoff-de-Matos

**Affiliations:** 1 Faculdade de Medicina, Departamento de Cirurgia Geral, Divisão de Cirurgia Vascular, Universidade Federal Fluminense, Niterói, Rio de Janeiro, 24220-002 Brasil; 2 Liv Care Centro Clínico, Niterói, Rio de Janeiro, Brazil; 3 Vascular Surgery Service, Hospital Ana Nery, Salvador, Bahia, Brazil; 4 Vascular Surgery Service, Hospital Universitário Clementino Fraga Filho (HUCFF), Universidade Federal do Rio de Janeiro (UFRJ), Rio de Janeiro, Rio de Janeiro, Brazil; 5 Afya Hospital Dia, Brasília, Distrito Federal, Brazil; 6 Image Department, Hospital Niterói Dor, Niterói, Rio de Janeiro, Brazil; 7 Vascular Surgery Service, Hospital São Luiz Anália Franco, São Paulo, SP Brazil; 8 Vascular Surgery Service, Hospital Niterói Dor, Niterói, Rio de Janeiro, Brazil; 9 Instituto Nacional de Traumatologia E Ortopedia, Rio de Janeiro, Rio de Janeiro, Brazil; 10 Faculdade de Medicina, Departamento de Medicina, Divisão de Nefrologia, Universidade Federal Fluminense, Niterói, Rio de Janeiro, Brasil

**Keywords:** Arteriovenous fistula, Balloon-assisted maturation, Drug-coated balloon angioplasty, Hemodialysis vascular access, High-pressure balloon angioplasty, Vascular access

## Abstract

**Purpose:**

To evaluate arteriovenous fistula maturation and 12-month patency in patients undergoing oversized balloon angioplasty with high-pressure balloons alone or with high-pressure balloons plus drug-coated balloons as part of endovascular treatment for arteriovenous fistula maturation failure.

**Material and Methods:**

This prospective, non-randomized study was conducted between 2022 and 2024 at four centers in Brazil. Patients with primary arteriovenous fistula failure underwent oversized balloon angioplasty with high-pressure balloons alone or with high-pressure balloons + drug-coated balloons. Vascular access primary and secondary patency rates were evaluated at 3, 6, and 12 months in 198 patients. The safety endpoint was the incidence of procedure-related complications. Exploratory analyses included the impact of demographic and clinical variables on primary patency rates.

**Results:**

Technical success was achieved in 99% of patients (196/198). At 12 months, vascular access primary patency was 57.8% for high-pressure balloons + drug-coated balloons vs. 41.3% for high-pressure balloons alone (*p* = 0.011). Twelve-month vascular access secondary patency rates were similar between groups (88.5% for high-pressure balloons + drug-coated balloons vs. 87.0% for high-pressure balloons alone, p = 0.69). Cox regression analysis identified oversized balloon angioplasty with drug-coated balloons and male sex as protective factors, reducing the 12 month risk of primary patency loss. In contrast, brachiocephalic arteriovenous fistulas and initial thrombosis were associated with an increased risk of patency loss.

**Conclusion:**

Oversized balloon angioplasty with drug-coated balloons improved primary patency compared to high-pressure balloons alone.

**Graphical Abstract:**

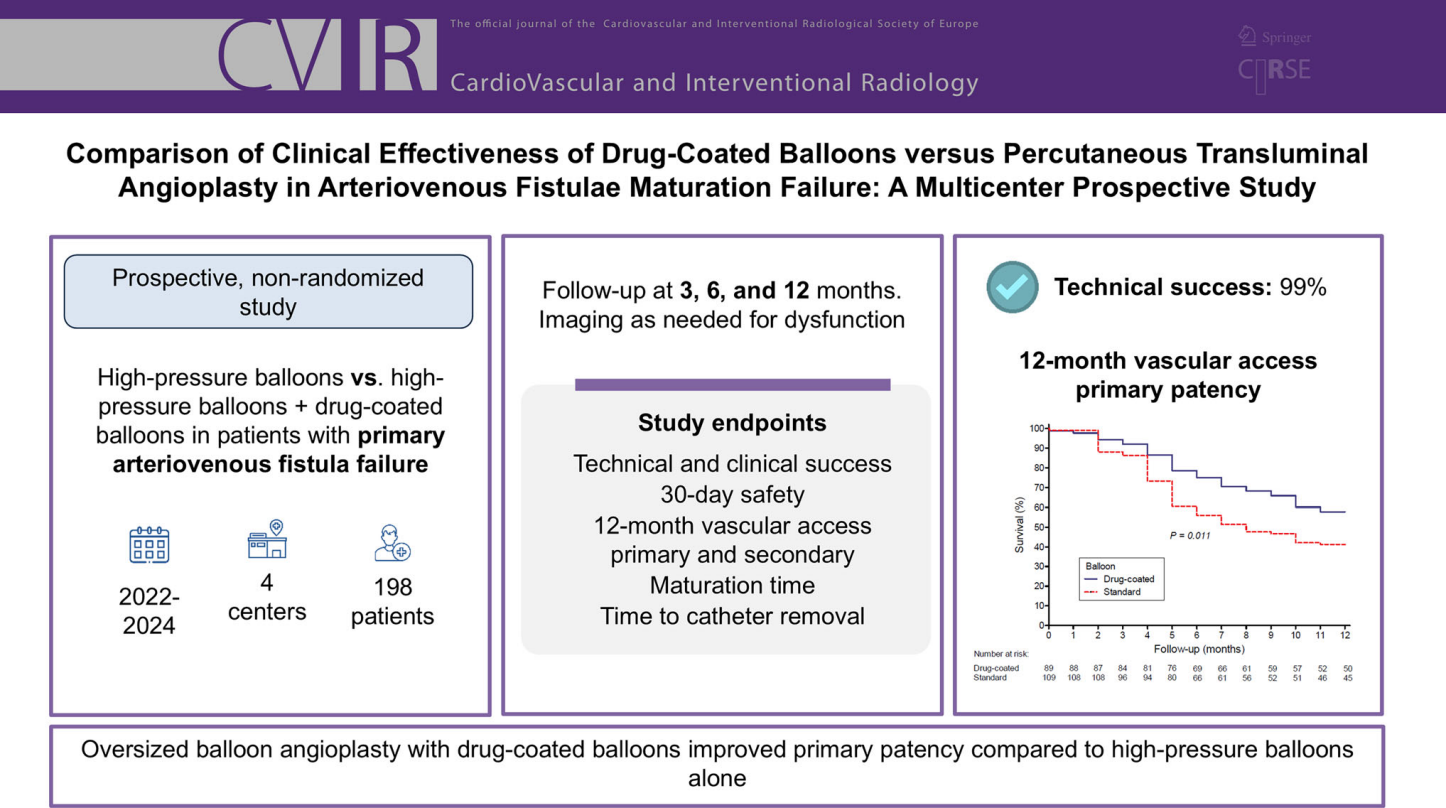

## Introduction

Maturation failure, often caused by neointimal hyperplasia leading to venous stenosis, affects up to 60% of newly created arteriovenous fistulas (AVF) [1] and results in inadequate blood flow and hemodialysis access dysfunction [2–4].

Balloon-assisted maturation (BAM) is an endovascular technique used to enhance AVF maturation in cases of inadequate development [5]. Oversized balloon angioplasty using high-pressure balloons (HPB) has been proposed as a strategy to improve BAM outcomes by more effectively expanding the venous lumen and minimizing vessel recoil, thereby enhancing fistula maturation and reducing times to catheter-free hemodialysis [6, 7].

Drug-coated balloons (DCBs) have been introduced as an adjunct to standard angioplasty. DCBs reduce restenosis by delivering antiproliferative agents like paclitaxel directly to the vessel wall, improving the patency of the AVFs compared with plain balloon alone [8–13].

Comparative evidence on the impact of oversized balloon angioplasty with HPBs alone versus HPB + DCBs on vascular access primary patency (VAPP) and maturation outcomes remains limited. This study addresses this gap by (1) evaluating vascular access primary and secondary patency at 3, 6, and 12 months; (2) assessing technical success, clinical success, and procedural safety; and (3) exploring demographic and clinical predictors of primary patency in patients undergoing oversized balloon angioplasty with HPBs alone or with HPB + DCB as part of endovascular treatment for AVF maturation failure.

## Material and Methods

### Study Design and Patient Selection

**Fig. 1:**
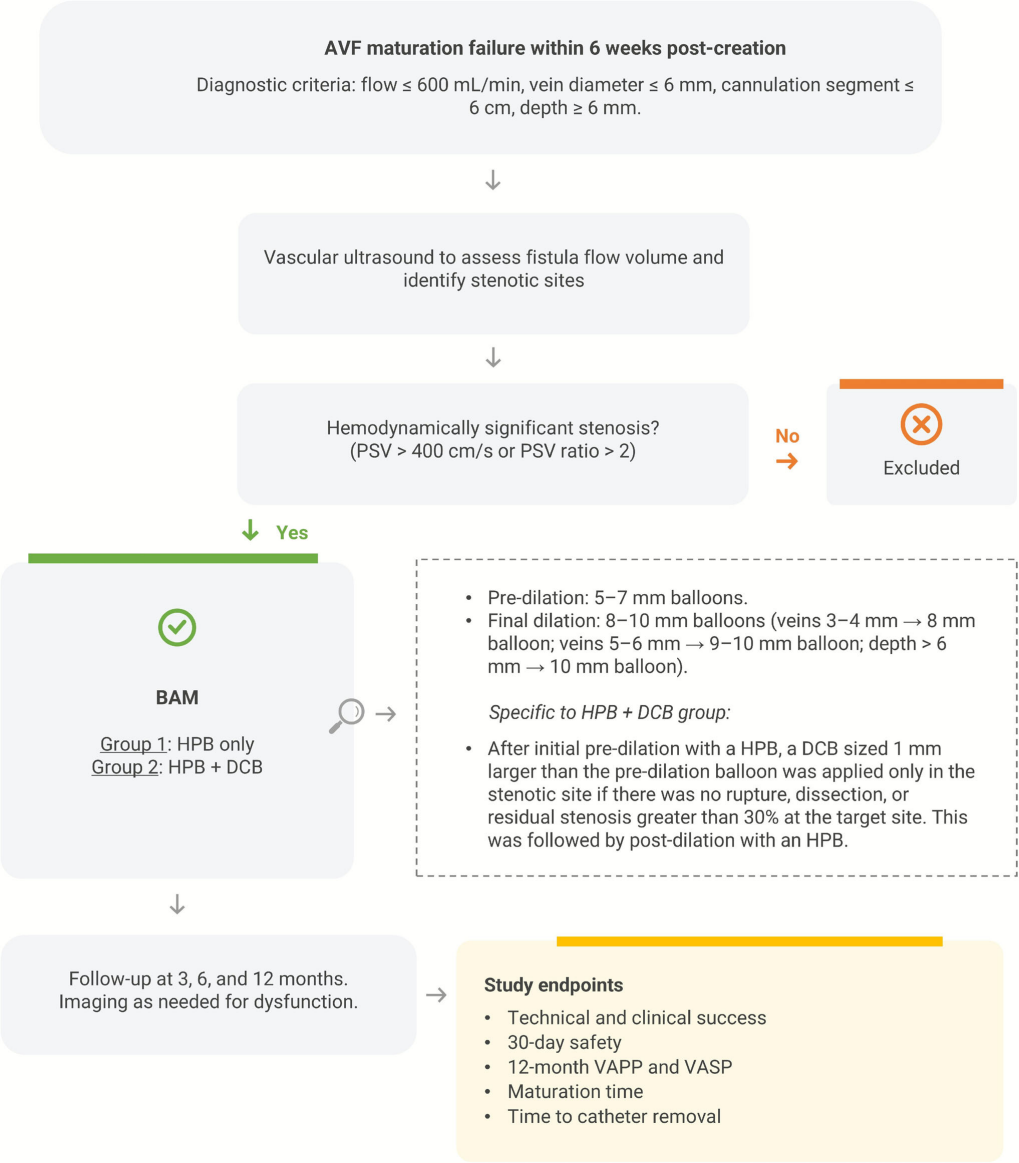
Study design AVF: Arteriovenous fistula; PSV: peak systolic velocity; BAM: balloon-assisted maturation; HPB: high-pressure balloon; DCB: drug-coated balloon; VAPP: vascular access primary patency; VASP: vascular access secondary patency

This is a prospective, non-randomized study performed between June, 2022 and March, 2024 at four Brazilian vascular access centers (one outpatient clinic and three hospitals). Figure 1 details the study protocol. Patients were eligible for inclusion in the study if they presented with primary AVF failure (defined as failure to achieve adequate maturation within six weeks post-creation – Fig. 1), associated with stenosis in the vascular access circuit, confirmed by vascular ultrasound. Patients with central venous stenosis were excluded from the analysis.

Patients were allocated to one of two treatment groups: (1) oversized balloon angioplasty with HPB alone (Conquest® [Bard® Peripheral Vascular, Inc. Tempe, Arizona, USA] or Mustang® [Boston Scientific, Marlborough, Massachusetts, USA]), or (2) oversized balloon angioplasty with HPB + DCB (Lutonix™ [BD, Franklin Lakes, New Jersey, USA] or Ranger [Boston Scientific, Marlborough, Massachusetts, USA]).

Because DCBs may reduce restenosis rates compared to conventional angioplasty balloons in failing AVFs [14], DCB angioplasty was prioritized whenever possible and applied to the stenotic segment responsible for non-maturation. However, due to heterogeneous insurance coverage in Brazil, group allocation was determined by payer approval: patients with coverage for DCBs underwent HPB + DCB angioplasty, whereas those without coverage were treated with HPB alone.

### Endovascular Technique

Two vascular surgeons and two interventional radiologists with more than 10 years of experience in vascular access performed the procedures in this study. The endovascular technique was performed as described by Harduin et al. (2021) [6]. Veins were pre-dilated with balloons measuring 5–7 mm in diameter, followed by final dilation with 8–10 mm diameter balloons, selected based on preoperative ultrasound measurements. Specifically, veins with a preoperative diameter of 3–4 mm were dilated with an 8 mm balloon. Those measuring 5–6 mm underwent final dilation with a 9–10 mm balloon. In patients in which the vein depth exceeded 6 mm, a 10 mm balloon was used for final dilation. The venous segment, including the stenotic area, was dilated progressively from the outflow tract to the anastomosis, where the final angioplasty was performed. This approach ensured optimal flow restoration while minimizing the risk of rupture from circuit pressurization. For procedures involving stenosis in the radial artery or anastomosis, smaller balloons were used initially (3–4 mm diameter balloon to dilate the radial artery and 5–6 mm to dilate the anastomosis). For patients treated with DCBs, the balloon was used as an intermediate step in the angioplasty sequence. After initial pre-dilation with a HPB, a DCB sized 1 mm larger than the pre-dilation balloon was applied only in the stenotic site if there was no rupture, dissection, or residual stenosis greater than 30% at the target site. The DCB was inflated for 3 min, after which final post-dilation was performed using an HPB. On average, three dilations with HPB (to burst pressure) were performed per patient, each lasting one minute. During dilation, a team member-controlled inflow by manually compressing the anastomosis to reduce the risk of vessel rupture.

### Outcome Measures

Technical success was defined as < 30% residual stenosis on post-procedure angiography [15]. Clinical success was defined as successful AVF cannulation without the need for reintervention within 30 days post-procedure [15]. Complications were evaluated within 30 days post-procedure and categorized according the modified CIRSE classification system [16].

Patients underwent routine follow-up at the outpatient vascular surgery clinic, with physical examinations and Duplex ultrasound scheduled at 3, 6, and 12 months post-procedure. Angiography was recommended if the AVF showed signs of dysfunction, such as difficult cannulation, prolonged bleeding, elevated venous pressure, diminished thrill, or increased pulsatility.

### Statistical Methods

The sample size required to compare the primary outcome, 12-month VAPP, was estimated assuming a patency rate of 65% in the DCB angioplasty group and 40% in the HPB angioplasty group. Assuming a two-sided α level of 0.05 and a statistical power of 80%, the standard formula for comparing two proportions yielded a required sample size of 61 patients per group. To account for an anticipated 25% loss to follow-up, the final adjusted sample size was increased to 82 patients per group, resulting in a total enrollment target of 164 patients.

Demographic and clinical characteristics of patients were summarized using descriptive statistics. Continuous variables were presented as means with standard deviations, and categorical variables were reported as frequencies and percentages. Comparisons between categorical variables were performed using the Chi-Square test, and continuous variables were analyzed using Student’s t-test.

Patency analysis in the intention-to-treat population was performed using the Kaplan- Maier method, and the results were compared using the log-rank test. Patients lost to follow-up at specified time points were not included in the analysis (the population at risk at each time interval is displayed on the x axis of the Kaplan–Meier curve). A Cox regression model estimated the hazard ratio for 12-month AVF patency loss in the intention-to-treat population.

All statistical analyses were performed using SPSS software (version 18.0, IBM, Armonk, NY, USA), and p-values < 0.05 were considered statistically significant.

## Results

### Patient Demographics and Clinical Characteristics

Table 1 summarizes the demographics and clinical characteristics of 198 patients (89 patients with HPB + DCB). The groups had similar characteristics.

**Table 1:** Patient demographics and clinical characteristics

Variable		HPB + DCB (n = 89)	HPB (n = 109)	*P*-value
Age (years) at the surgery date; (mean ± SD)		62.7 ± 13.6	61.3 ± 14.3	0.654
Male sex; n (%)		53 (60%)	62 (57%)	0.632
Hypertension; n (%)		72 (81%)	88 (81%)	0.952
Diabetes mellitus; n (%)		48 (54%)	61 (56%)	0.754
Current smoker; n (%)		15 (17%)	20 (18%)	0.879
Stroke; n (%)		6 (07%)	9 (08%)	0.798
Coronary heart disease; n (%)		18 (20%)	23 (21%)	0.874
Pre-operative vein diameter (mm); (mean ± SD)		4.26 ± 1.88	4.29 ± 1.84	0.844
AVF type; n (%)	Brachiocephalic	36 (40%)	44 (40%)	0.981
	Radiocephalic	36 (40%)	36 (33%)	0.272
	Basilic vein transposition	14 (16%)	28 (26%)	0.071
	Ulnar basilic	03 (4%)	1 (1%)	0.319
Initial thrombosis; n (%)		12 (13%)	25 (23%)	0.062
Location of stenosis; n (%)	Juxta-anastomosis	49 (55%)	55 (50%)	0.478
	Venous outflow	04 (5%)	09 (08%)	0.394
	Middle third of the vein	10 (11%)	15 (14%)	0.656
	Multiple sites	25 (28%)	28 (26%)	0.897
	Radial artery	01 (1%)	02 (2%)	0.983
Age of the AVF at the time of maturation (weeks); (mean ± SD)		6.8 ± 2.26	10.1 ± 3.36	< 0.0001
Time to use the AVF after oversized balloon angioplasty (days); (mean ± SD)		4.63 ± 3.67	5.38 ± 4.13	0.178
Time to catheter removal after oversized balloon angioplasty (days); (mean ± SD)		12.72 ± 3.99	13.81 ± 3.92	0.065
Number of interventions per patient in 12 months; (mean ± SD)		1.31 ± 0.45	1.57 ± 0.61	0.001

AVF, arteriovenous fistula; DCB, drug-coated balloon; HPB, high-pressure balloon; SD, standard deviation

### Procedural and Safety Outcomes

Technical and clinical success was achieved in 99% of patients (196 out of 198 who underwent the BAM procedure). Two patients experienced complete AVF rupture, requiring access ligation; five had residual stenosis greater than 50% and required stent placement for maturation, and three developed pseudoaneurysms, which were treated with covered stents during the initial maturation procedure. In patients who required stents or covered stent deployment, the maturation was achieved, and the cannulation was performed successfully in the native vein or through the stents if necessary. Grade 1a complications were similar between the groups (14.6% for HPB alone vs. 13.4% for HPB + DCBs; *p* = 0.751). Grade 2 complications occurred in 1.5% of the patients (3/198). Two patients (one in each group) presented with grade 3a complications and required a new AVF creation.

During follow-up, 58 patients required one additional procedure, and 28 patients required two additional procedures to maintain patency. These procedures were performed in 44 brachiocephalic AVFs, 25 radiocephalic AVFs, 16 basilic vein transpositions, and one ulnar-basilic AVF.

Seventeen patients did not complete the 12-month follow-up. Among these patients, two received a kidney transplantation and three required AVFs ligation (one due to a grade 4 hemodialysis access-induced distal ischemia [HAIDI] developed later at six months post-procedure, and two due to infection at the cannulation site, occurring at five and six months of follow-up). The other 12 patients died from cardiovascular causes not related to vascular access complications.

### Performance Outcomes

**Fig. 2:**
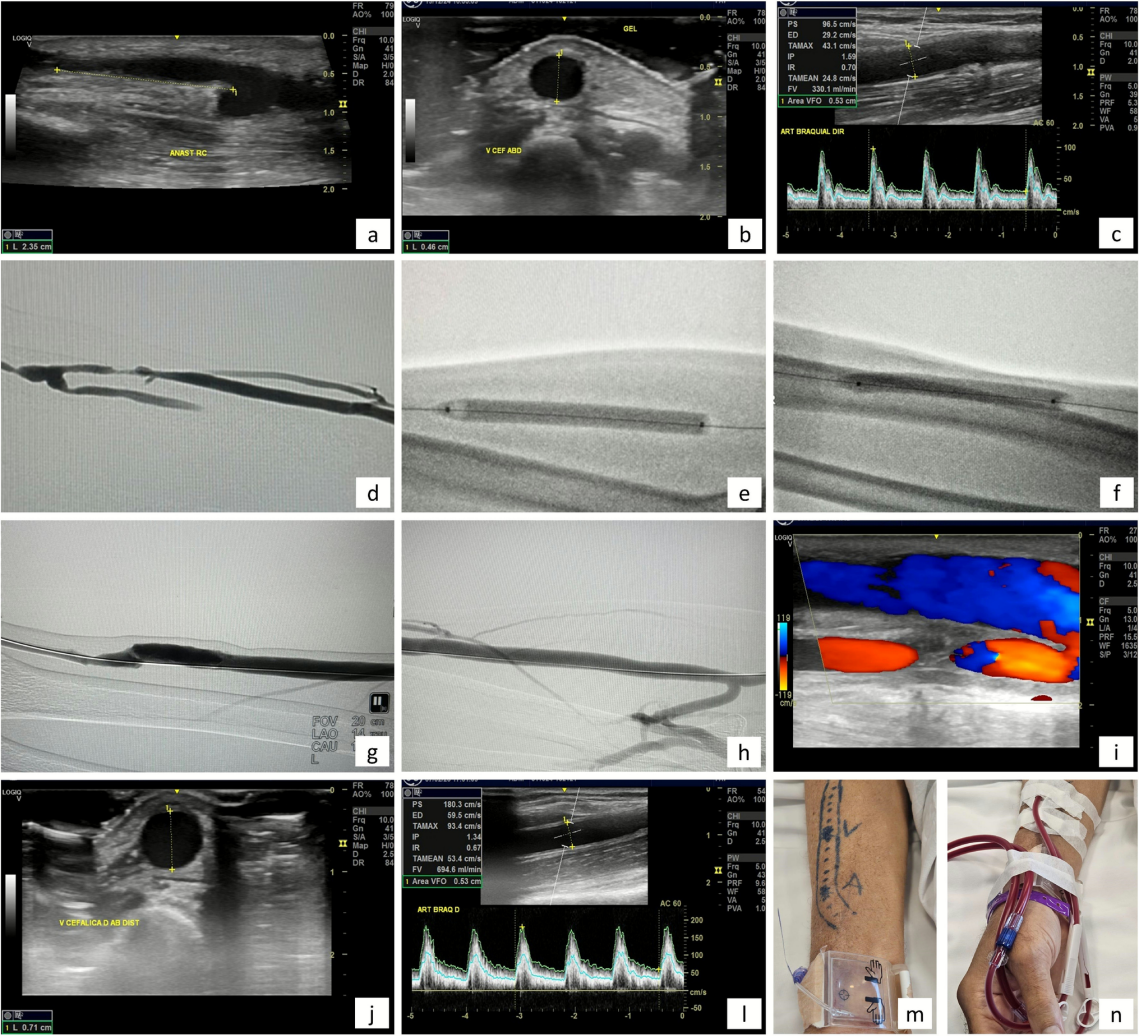
Example of procedural outcomes in a patient with a left radiocephalic fistula and juxta-anastomotic stenosis. **a** Left radiocephalic fistula showing juxta-anastomotic stenosis. **b** Non-matured cephalic vein with a diameter of 4.5 mm. **c** Pre-procedural brachial artery flow volume measured at 330 mL/min, confirming non-maturation. **d** Intraoperative angiography showing severe juxta-anastomotic stenosis and inadequate venous diameter (radial access with 6F sheath). **e** Venous angioplasty initiated at the outflow segment (elbow). **f** Angioplasty performed at the juxta-anastomotic (inflow) segment. **g** Final angiographic result of the inflow and cannulation zones after angioplasty. **h** Final angiography showing the outflow segment after successful maturation. **i** Post-procedure Doppler ultrasound of the juxta-anastomotic segment. (**j**) Cephalic vein with appropriate diameter for cannulation immediately post-procedure. **l** Post-procedural brachial artery flow volume increased to 694 mL/min. **m** External view of the matured fistula. **n** Fistula successfully cannulated 12 h after the procedure

Figure 2 displays the procedural outcomes of an example patient. The average flow volume in the fistula before the procedure was 341 ml/min (range 122 – 543 ml/min), increasing to 984 ml/min (range 619 – 1879 ml/min) after the procedure.

**Fig 3:**
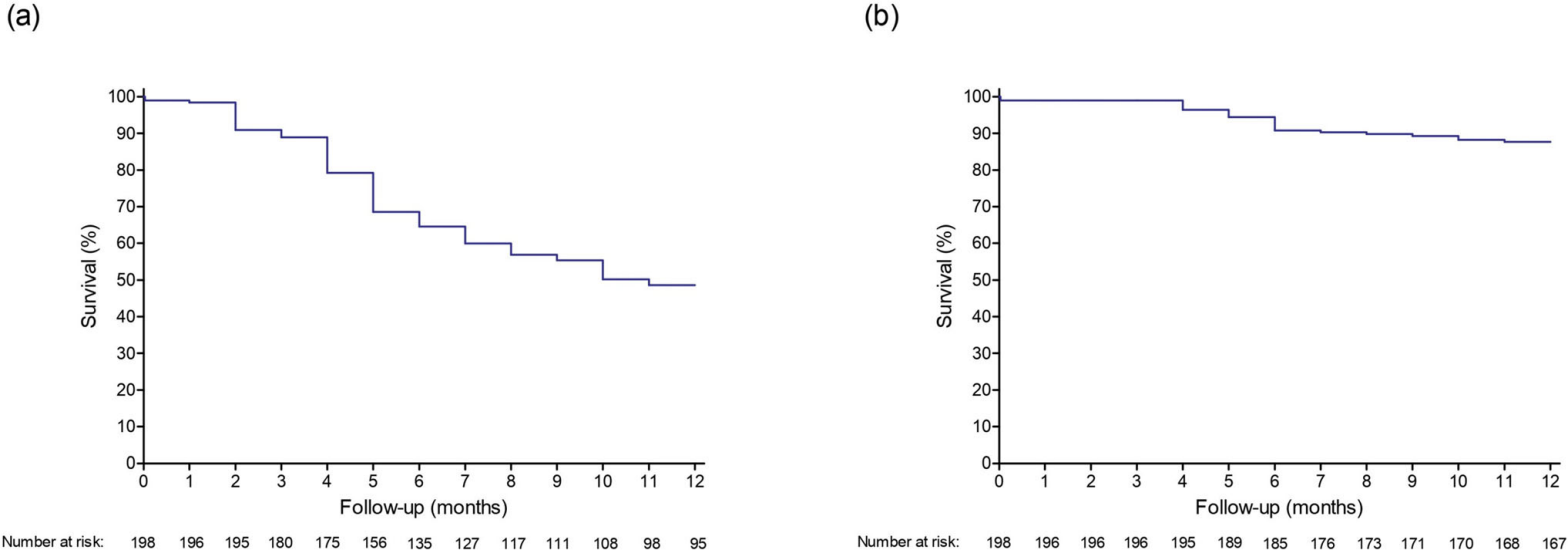
Kaplan–Meier survival curves for vascular access primary patency (Panel a) and vascular access secondary patency (Panel b) in the intent-to-treat population (n = 198)

No statistically significant differences were observed between the groups regarding the time to use the AVF after oversized balloon angioplasty (5.38 days for HPB alone vs. 4.63 days HPB + DCBs; p = 0.178) or the time to remove the catheter following the procedure (13.81 days vs. 12.72 days; p = 0.065) (Table 1). At 12 months, the VAPP rate was 48.7% and the vascular access secondary patency (VASP) rate was 87.7% (Fig. 3).

**Fig. 4:**
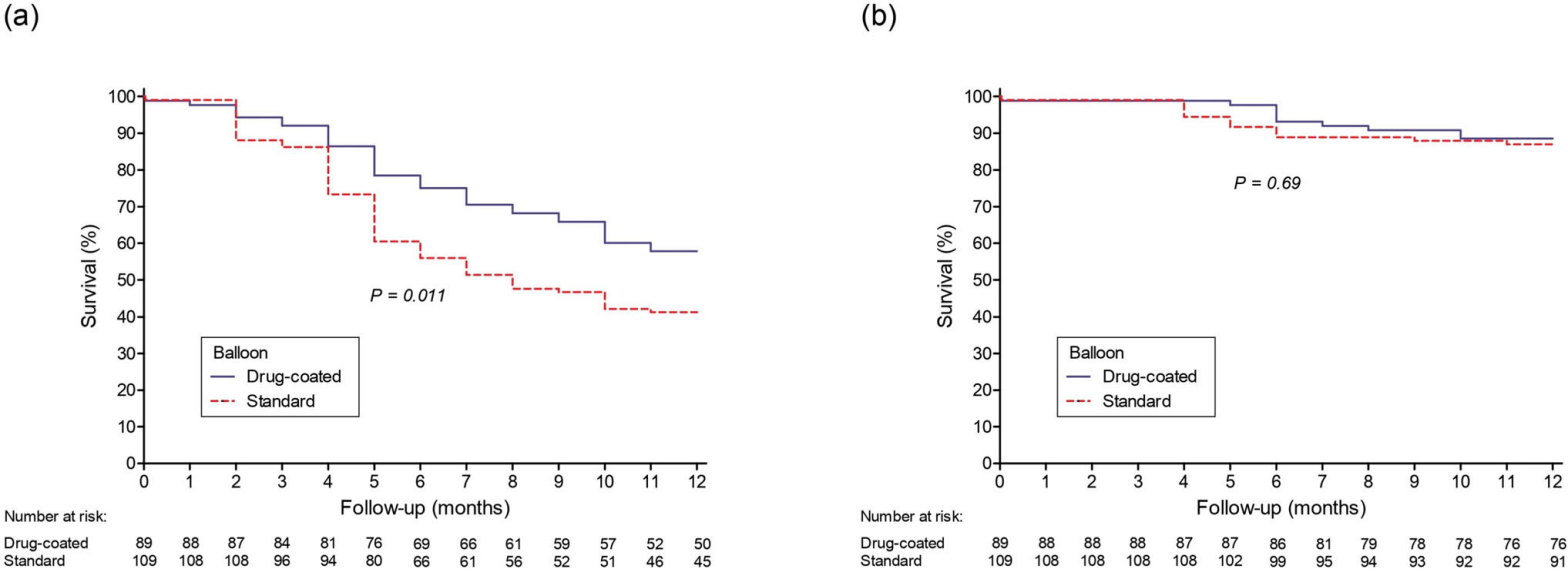
Kaplan–Meier survival curve illustrating vascular access primary patency (panel a) and vascular access secondary patency (Panel b) rates over time, stratified by treatment group in the intention-to-treat population

As shown in Fig. 4a, at 12 months, VAPP was significantly higher in the group subjected to oversized balloon angioplasty with HPB + DCBs vs HPB alone (57.8% vs. 41.3%, p = 0.011). However, no significant difference between groups was observed in VASP (88.5% for HPB + DCBs vs. 87% for HPB alone, *p* = 0.69) (Fig. 4b), although statistically more interventions were necessary to maintain patency in the HPB alone group (1.57 vs. 1.31, p = 0.001).

As shown in Table 2, the DCBs use (HR 0.60, 95% CI 0.39 – 0.91, p = 0.015) and male gender (HR 0.50, 95% CI 0.33 – 0.76, *p* = 0.001) were significantly associated with a lower risk of VAPP loss. In contrast, having a brachiocephalic AVFs (HR 1.51, 95% CI 1.15 – 1.99, *p* = 0.003) and initial thrombosis at presentation (HR 1.90, 95% CI 1.18 – 3.07, *p* = 0.009) were associated with a higher risk of VAPP loss.

**Table 2:** Cox regression analysis for prediction of 12-month vascular access primary patency failure (intention-to-treat population)

Variable	Unadjusted model			Adjusted model		
	HR	95% CI	*P*-value	HR	95% CI	*P*-value
Drug-coated balloon	0.61	(0.40 – 0.91)	0.015	0.60	(0.39 – 0.91)	0.015
Male gender	0.46	(0.31 – 0.69)	< 0.001	0.50	(0.33 – 0.76)	0.001
Age (years)	1.00	(0.99 – 1.01)	0.99	-	-	-
Diabetes	1.01	(0.69 – 1.50)	0.95	-	-	-
Anatomic site of AVF						
Forearm (ref.)						
Brachiocephalic	1.72	(1.10 – 2.67)	0.017	1.51	(1.15 – 1.99)	0.003
Basilic transposition	1.08	(0.61 – 1.90)	0.80	0.78	(0.54 – 1.11)	0.17
Venous diameter						
4 mm (ref.)						
< 4 mm	1.66	(1.21 – 2.28)	0.002	1.36	(0.91 – 2.04)	0.13
> 4 mm	0.52	(0.34 – 0.81)	0.004	0.64	(0.38 – 1.05)	0.079
More than one stenotic segment	2.04	(1.36 – 3.07)	0.001	1.63	(0.99 – 2.67)	0.054
Initial thrombosis	2.07	(1.32 – 3.27)	0.002	1.90	(1.18 – 3.07)	0.009
Balloon diameter > 8 mm	0.65	(0.42 – 0.99)	0.047	0.73	(0.44 – 1.22)	0.23

HR, hazard ratio; CI, confidence interval; AVF, arteriovenous fistula

## Discussion

The present study showed the feasibility and efficacy of the oversized BAM technique to treat primary AVF maturation failure in a large prospective and multicenter cohort. Moreover, the additional use of a drug-coated balloon, as an intermediate step in the procedure, significantly increased the 12-month VAPP (58% vs 41%), reducing the need for new interventions, whereas the 12-month VASP rates reached almost 90% in both groups.

A recent systematic review and meta-analysis [5], with a pooled analysis of 13 studies comprising 1,427 patients with non-matured AVFs, reinforced the efficacy and safety of the BAM technique in salvaging non-matured AVFs. The oversized BAM technique was first described by Harduin et al. [6], in a retrospective single-center study, in which they found primary and secondary patency of 51% and 90%, respectively, demonstrating its feasibility and efficacy in accelerating AVF maturation and reducing catheter dependency. More recently, Juneja et al. [7] reproduced the technique and confirmed the short-term benefits of oversized BAM in optimizing AVF maturation. Their study reported an AVF maturation rate of 97% in patients treated with larger balloons (≥ 7 mm) compared to 88% in those treated with smaller balloons. Additionally, at 60 days after the procedure, the incidences of catheter-free dialysis for patients treated with larger and smaller balloons were 83% and 67%, respectively. Of note, drug-coated balloons were not used in either of these previous studies. Our study advances existing knowledge by comparing oversized BAM using HPBs alone versus HPB + DCBs.

The present study reported a 60% reduction in the adjusted hazard ratio of VAPP loss within one year when oversized balloon angioplasty with DCBs was compared to HPB alone. The superior performance of DCBs can be attributed to their ability to deliver antiproliferative agents that inhibit neointimal hyperplasia, a key driver of AVF stenosis and failure [8, 10, 17], whereas HPB only addresses the mechanical stenosis.

Serious complications were infrequent, with two patients experiencing a complete AVF rupture requiring access ligation, and three developing pseudoaneurysms. The incidence of complications is compatible with previous studies of the conventional BAM technique [5, 18, 19]. Nonetheless, dilation of the entire venous segment to accelerate AVF cannulation raises concern for endothelial injury and subsequent stenosis in healthy venous segments. Despite the extensive mechanical intervention in this study, the VAPP outcomes were comparable or even superior to those reported in previous trials with angioplasty restricted to focal stenoses [20, 21]. Furthermore, most of the restenosis in our patients occurred at the initial stenotic site responsible for the primary maturation failure rather than in previously normal segments.

Beyond safety considerations, the primary clinical advantage of the oversized BAM technique is the ability to accelerate functional access. Conventional strategies often require 5 weeks for an AVF to become usable, with a longer period of time to remove the catheter [18, 19, 22, 23]. In the present study, the mean time to first AVF cannulation after the oversized BAM procedure was 5 days, and the catheter was removed on average less than 2 weeks after the intervention. With the use of the oversized BAM technique, AVFs could be cannulated earlier, but no significant differences between groups were observed regarding the time to use the AVF or catheter removal time. The early high successful maturation rate in both groups, regardless of DCB use, could be explained by the primary role of the mechanical correction of the stenosis by the HPB, whereas the balloon paclitaxel delivery would have a lasting effect, preventing restenotic lesions and increasing the VAPP rate.

Strengths of this study include its prospective design and the direct comparison between oversized balloon angioplasty with DCBs and HPB alone. However, limitations should be acknowledged. First, the follow-up period, while sufficient to assess mid-term outcomes, does not provide long-term durability data. Second, because the use of DCBs depended on healthcare insurance approval, patients did not have uniform access to the two treatment strategies. As the criteria for reimbursement authorization for DCB use vary between insurance companies, this introduces a potential source of selection bias. Anyway, the baseline demographic and clinical characteristics of the groups were similar, and the reduced risk of 12-month VAPP loss associated with DCB use persisted after adjustment for potential confounder variables. Third, although intention-to-treat analyses were performed and all major complications were incorporated into the safety assessment, the cohort included a heterogeneous mix of patients with juxta-anastomotic stenosis, venous stenosis, arterial stenosis, and complete thrombosis. This type of heterogeneity is common across BAM studies and reflects real-world clinical practice; however, it may affect the generalizability of patency outcomes. Notably, more than half of non-maturation cases in our cohort were due to juxta-anastomotic stenosis, historically the most frequent cause of early AVF failure [24, 25], which helps contextualize the population studied.

## Conclusion

Our findings suggest that oversized balloon angioplasty with HPB + DCB improves VAPP compared with oversized balloon angioplasty with HPB alone, addressing a key challenge in dialysis access maintenance.
